# Bacteriology in Its Relation to Veterinary Science1Read before the Keystone Veterinary Medical Association, Philadelphia, Pa., December 8, 1896.

**Published:** 1897-02

**Authors:** M. P. Ravenal


### BACTERIOLOGY IN ITS RELATION TO VETERINARY SCIENCE.[1]

By M. P. Ravenal, M.D.

(Concluded from page 14.)

It would be most tempting to speculate on the possible achievements of the future and to dwell longer on what has been already accomplished; but the object of this address, as promised, is to give a short and practical talk directed mainly to pointing out to you how the general practitioner may best assist the specialist in bacteriology in his work for our future benefit. It is known to you all, I believe, that through the efforts of our able State Veterinarian a bacteriological laboratory has been established at the University of Pennsylvania by the State Live-stock Sanitary Board, and it is our wish and object to make the work of this laboratory of benefit to the community and State in general as well as to advance our knowledge of the cause and prevention of disease. The specialist in his laboratory has but little opportunity of seeing cases other than experimental ones or of knowing even what is going on among practitioners, and it is here that you can aid us greatly, and in return we will do all we can to aid you. While it requires much time and attention to do bacteriological work, and for that reason the science will probably always remain in the hands of specialists to a great extent, every practitioner can readily acquire enough of the technique and the principles on which it is

[1] Read before the Keystone Veterinary Medical Association, Philadelphia, Pa., December 8, 1896.

founded to enable him to secure cultures and specimens which can then be worked up by a specialist in the laboratory. I lay stress on this point because much valuable material goes to waste through improper methods of collection, and the bacteriologist is likely to be blamed for what is not his fault. A few words on this point may not be amiss. The apparatus required for making cultures is not elaborate. A stout platinum needle mounted in aluminum, an alcohol lamp, and a few culture-tubes are all that will ordinarily be required. Cases are now put up containing these articles, and may be obtained from the makers of surgical instruments. In addition to this a few sterilized pipettes made from glass-tubing are often useful. Beyond these things nothing is required except the instruments every practitioner always has—a scalpel or razor. If the culture or specimen is to be taken post mortem, let it be as soon after the death of the animal as possible. The internal organs especially soon become invaded by intestinal organisms after death, and our work may thus be hindered or even rendered negative entirely. The surface of the organ from which the culture is to be made must be seared with a hot piece of metal and the needle plunged through the sterile area thus formed. Blocks of tissue for the cutting of sections must also be taken as soon after death as possible and preserved in 95 per cent. alcohol, or, if it can be obtained, better still in absolute alcohol, when it is to be examined for bacteria. Blood, pus, urine, etc., are best collected by means of the pipettes as a rule, or where larger quantites are wished a sterile canula may be used, the fluid being drawn into sterile, glass-stoppered vials. The cardinal principle always is to use sterile apparatus and implements, this end being best attained by the use of heat, either dry or moist. As a rule, large quantities of material are not required. The sterilization of implements and receivers is readily done in your own homes by boiling in water for fifteen to twenty minutes or exposure in the oven of a stove for one hour or more. In many houses the Arnold steam sterilizer may now be found, making the process still more easy. Do not think that the elaborate apparatus often seen in laboratories is necessary to accomplish your ends. The principles once mastered, efficient apparatus may often be improvised from the ordinary household utensils. (One of our great American surgeons was in the habit of saying that he treated fractures with "splints and common sense".) Success in bacteriological work depends largely on this latter element, and I may add one more—attention to details, remembering that a chain is no stronger than its weakest link.

Such is an outline of the rules to be observed for all cases, though they may be more or less modified according to the disease we are investigating. For instance, material from the intestines or the discharges from the nostrils in a disease like glanders will always be contaminated, and no precautions as to the sterility of our containing-vessels will prevent it; but even here we should still be strict, for otherwise we could not be sure of the source of the organisms found. Always, whatever the specimen may be, it must be sent to the laboratory as rapidly as possible after collection, and unless it is preserved in alcohol should be kept on ice as a rule. I have had the brain of an animal suspected of rabies sent to me in such an advanced state of decomposition that no positive results were possible, the inoculated animals dying as the result of infection from the putrid mass rather than from any hydrophobic virùs it may have contained.

The study of the rarer diseases is very attractive, and in some ways seems to offer the greatest rewards for our search, but such is not the case. They are not to be neglected, but it is the widespread diseases that will yield the best results in most cases. Tuberculosis is unquestionably the most important, general, and destructive disease with which we have to deal, whether in man or in the domestic animals. Too much study cannot be given to it, either in the laboratory or in its clinical manifestations. In spite of the work which has been done on it, the question of transmissibility from animals to man is still somewhat unsettled, there being those who hold the opinion that there are two distinct diseases and probably non-communicable from one species to the other, while by others the cow is regarded as being the most potent factor in the causation of human tuberculosis; and I have heard the opinion expressed that human tuberculosis would disappear if the use of the cow and its products could be stopped. The truth lies probably in the middle ground. As to the transmission by milk there can be but little doubt, I think; but it seems necessary that there must be tubercular involvement of the lymphatics and udders. Tuberculosis may remain localized for years and do apparently but little harm, and then all at once become generalized. Hence an animal with even the smallest manifestation of the disease is a menace to the whole herd and to those using the products of that herd.

I cannot too warmly advocate the stand taken by the authorities in this State as to the slaughter of tuberculous animals, and I regard it as the duty of all who are interested in public hygiene as applied in its broadest sense to support those who have the carrying out of these laws in their hands. It must be a work of education by those of us who have had the advantages of study and observation. While I do not anticipate the disappearance of the disease entirely by the enforcement of these laws, I do hope for a vast improvement in the health of our people as well as of our herds.

The diagnosis of tuberculosis is not always easy from clinical symptoms, nor from post-mortem examinations; but fortunately in tuberculin or Koch's lymph, as it was called, we have an almost infallible means of detecting the disease. The bacteriologist with his microscope may not be able to demonstrate the presence of the bacillus in the cheesy masses so characteristic of the tubercular process, yet the inoculation of a susceptible animal with this material will cause the disease. This fact has given rise to doubt in the minds of some who were inclined to doubt the causative agency of the tubercle bacillus, but is now explained by the formation of spores, by the tubercle bacillus in the tissues, which need only to be transplanted to suitable soil to vegetate and multiply. Cultures of the bacillus are reported to have been obtained from the dust of rooms and stables in which it could not be found by the microscope. The difficulty of cultivating the tubercle bacillus makes culture-experiments of somewhat doubtful value, and the intraperitoneal inoculation of susceptible animals, such as guinea-pigs, offers very much surer results. Milk or pus, for instance, in which the bacillus cannot be demonstrated by the microscope, will often cause tuberculosis on inoculation.

A volume might easily be written on this subject alone, but I must leave it now with one more suggestion, applicable to all other diseases as well. I refer to the collection of statistics and observations. In this way the practitioner can do an immense amount of good in extending our knowledge, and from his observations laboratory experiments can be mapped out.

There are two other rather common diseases met with by the veterinarian in which an early and certain diagnosis is of great importance—anthrax and glanders. Diagnosis of anthrax is not usually difficult, and the inoculation of a mouse or guinea-pig with a little blood from the suspected animal will make the matter clear. The diagnosis derives its importance from the necessity of stopping an epidemic outbreak, which can be done by the use of vaccines, as well as from a sanitary standpoint in the disposal of the carcass. The anthrax bacillus in the growing stage is not hard to kill, but forms most resistant spores, which retain their vitality for long periods of time. These spores are never formed in the body during life, but may be present in the bloody discharges. After death when the tissues are opened and oxygen admitted spores may form. It is taught in some text-books that at the depth of seven or eight feet the temperature of the earth is so low as to prevent the formation of spores, and that burying the animal at this depth is a safeguard against future infection. Actual thermometric observations on bodies at this depth show that the changes due to decomposition develop heat enough to allow of the formation of spores. Pasteur further showed that these spores might be brought to the surface by earth-worms. This has been denied by Koch and his followers, but the experiments of Pasteur appear to me to be conclusive. We have two rapid and sure ways of diagnosticating glanders—by mallein and by the inoculation of guinea-pigs after the method of Strauss. Mallein is a glycerin extract of the bacillus mallei of glanders bacilli, for the introduction of which we are indebted largely to Dr. Leonard Pearson, who was one of the first to make an experiment with it. Its employment is now as well-recognized a procedure as is the use of tuberculin, to which it is closely allied so far as its manufacture and use are concerned. Strauss's method depends on the selective action of the glanders bacillus for the testicle of the guinea-pig when inoculated into the peritoneal cavity. If the culture be virulent, a suppurative orchitis is set up by the third or fourth day, and the diagnosis is absolute. Rupture of the testicle takes place later, and the animal shows general infection.

Among the less understood, but unfortunately too common diseases, I may mention osteoporosis and meningitis as being of peculiar interest. The general remarks as to collection of material for examination apply in these cases. The diseased tissues and the general circulation seem to offer the best material for study, and cultures should be made from these promptly. So far results have not been satisfactory, but there is no reason to give up the fight. Each year we improve our methods of technique and culture, and there is strong ground for hope that persistent effort will soon be rewarded by the elucidation of the etiology of these diseases.

I have tried, gentlemen, to point out succinctly how we may work together; but let me ask you not to depend too much on the specialist. Many of the methods described are of easy application, and each practitioner can do much for himself, and derive from them in consequence much more good than he would from work done by another and the results given to him.

